# Experimental study and parameters optimization of microalgae based heavy metals removal process using a hybrid response surface methodology-crow search algorithm

**DOI:** 10.1038/s41598-020-72236-8

**Published:** 2020-09-15

**Authors:** N. Sultana, S. M. Zakir Hossain, M. Ezzudin Mohammed, M. F. Irfan, B. Haq, M. O. Faruque, S. A. Razzak, M. M. Hossain

**Affiliations:** 1 Department of Computer Science, College of Computer Science and Information Technology, Imam Abdulrahman Bin Faisal University, Dammam, Saudi Arabia; 2 Department of Chemical Engineering, College of Engineering, University of Bahrain, Zallaq, Kingdom of Bahrain; 3 Department of Petroleum Engineering, King Fahd University of Petroleum and Minerals, Dhahran, Saudi Arabia; 4 Department of Chemical Engineering, King Fahd University of Petroleum and Minerals, Dhahran, Saudi Arabia

**Keywords:** Biotechnology, Chemical biology, Computational biology and bioinformatics, Environmental sciences, Materials science, Mathematics and computing

## Abstract

This study investigates the use of microalgae as a biosorbent to eliminate heavy metals ions from wastewater. The *Chlorella kessleri* microalgae species was employed to biosorb heavy metals from synthetic wastewater specimens. FTIR, and SEM/XRD analyses were utilized to characterize the microalgal biomass (the adsorbent). The experiments were conducted with several process parameters, including initial solution pH, temperature, and microalgae biomass dose. In order to secure the best experimental conditions, the optimum parameters were estimated using an integrated response surface methodology (RSM), desirability function (DF), and crow search algorithm (CSA) modeling approach. A maximum lead(II) removal efficiency of 99.54% was identified by the RSM–DF platform with the following optimal set of parameters: pH of 6.34, temperature of 27.71 °C, and biomass dosage of 1.5 g L^−1^. The hybrid RSM–CSA approach provided a globally optimal solution that was similar to the results obtained by the RSM–DF approach. The consistency of the model-predicted optimum conditions was confirmed by conducting experiments under those conditions. It was found that the experimental removal efficiency (97.1%) under optimum conditions was very close (less than a 5% error) to the model-predicted value. The lead(II) biosorption process was better demonstrated by the pseudo-second order kinetic model. Finally, simultaneous removal of metals from wastewater samples containing a mixture of multiple heavy metals was investigated. The removal efficiency of each heavy metal was found to be in the following order: Pb(II) > Co(II) > Cu(II) > Cd(II) > Cr(II).

## Introduction

Water pollution by heavy metals (e.g., Pb, Cu, Cd, Co, Cr, Zn, Ni) is a major environmental problem that poses severe hazards for both animal and human lives^1,2^. Exposure to these toxic metals may cause anemia, brain damage, liver and kidney diseases, interruption of vitamin D metabolism in children, and in serious cases, death ^3–5^. The primary origins of heavy metals are milling, textiles, mining, pigments, plastics, electroplating, metallurgical processes, and surface finishing industries ^6,7^. Generally, heavy metals are difficult to degrade in the natural environment. They have a high affinity to assemble in both the food chain and human body. As such, it is essential to remove heavy metals from both the wastewater and potable water streams in order to secure a healthy environment.

There are several alternative technologies available for eliminating heavy metals from wastewater, including electrocoagulation, precipitation, membrane filtration, ion exchange, advanced oxidation, reverse osmosis, evaporation, and adsorption. Commonly used adsorbents include chitosans, zeolites, nanoadsorbents, clay soils, barks, coconut-based materials, olive oil waste, and agricultural peels ^8–11^. Unfortunately, all of the present processes suffer from different types of problems including high energy consumption, low energy efficiency, substantial operation and maintenance costs, inadequate elimination, and generation of toxic waste. Conversely, the biosorption process via active or dead organisms/biomasses is a cost-effective method for the removal of heavy metals from industrial wastewater ^10–12^. In this approach, the biomass serves as a substrate with a range of functional groups with a nearly consistent distribution of binding sites. Low cost, high biosorption efficiency, superior selectivity, fewer biosorbents, and no toxic byproducts are some of the major merits of this technique. It is evident that a dead biomass is even more potent than a living biomass in eliminating heavy metals. Dead biomasses are normally available as waste or byproducts and do not require any nutrients or environmental conditions. In the past decade, most research on this topic has attempted to identify effective and readily available dead biomasses or biosorbents for eliminating heavy metals ^13–16^. Typically, these biosorbents include algae, fungi, yeast, bacteria, and agricultural waste ^13,15–18^. Surprisingly, there are only few studies available in the literature dealing with heavy metals removal using microalgae, which grows very rapidly and can act as a bisorbent for heavy metals.

The present study deals with the use of the *Chlorella kessleri* microalgae species to remove heavy metals from wastewater streams. The surface layers of microalgae are comprised mainly of lipids, polysaccharides, and proteins with many functional groups (e.g., –COOH, –PO_4_, –OH, –SO_4_ –NH_2_, –SH) that perform crucial roles in metal biosorption. The biosorption mechanism using algae is mainly an ion-exchange reaction. Initially, heavy metals ions are free in aqueous solution and light metals ions (e.g., Ca^2+^, Na^+^, Mg^2+^, K^+^) adhere to the functional groups. The ion-exchange reaction between metals ions and algal biomasses can be expressed as ^19^:

1 $$\left( {\text{L } - \text{ biomass}} \right) + {\text{M}}^{{{2} + }} \leftrightarrow \, \left( {\text{M } - \text{ biomass}} \right) + {\text{L}}^{{{2} + }}$$

where M^2+^ and L^2+^ denote heavy and light metals ions, respectively.

The biosorption efficiency of microalgae mainly relies on several independent variables: temperature, pH, metal dose, biomass dose, and residence time. To accurately assess the process, it is necessary to determine the individual impacts of these factors, along with the interaction impacts within the parameters of the response (i.e., the biosorption efficiency). Hereof, the response surface methodology (RSM) is a robust statistical tool for explaining the relationships among independent factors with dependent variables ^20–23^. The Box–Behnken design (BBD) (a type of RSM) reduces the number of laboratory trials and anticipates responses with high precision ^20,24^. Several studies have investigated RSM-based biosorption of heavy metals using microalgae ^25–28^. Although the RSM technique is efficient, it may provide only locally optimal solutions ^29^. Therefore, it may not provide a truly optimal solution. To overcome this drawback, it is preferable to use a globally or truly optimal solution.

Several well-known algorithms employed for global optimization such as the genetic algorithm (GA)^30^, simulated annealing (SA) ^31^, particle swarm optimization (PSO)^32^, ant colony optimization (ACP) ^33^, harmony searches (HS) ^34^, and others. However, the major bottleneck of these methods is the use of a high number of tuning parameters which make the processes labor-intensive. Contrarily, crow search algorithm (CSA), a metaheuristic optimization algorithm, is capable to overcome this drawback ^35^. It provides the global optimization of independent variables in different areas, including engineering research ^36–39^.

The present study pursues the optimization of biosorption process parameters (i.e., initial solution pH, temperature, and biomass dose) on Pb(II) elimination efficiency using RSM, separately articulated with a desirability function (DF) and the CSA. The DF technique is utilized widely for parameter optimization ^40,41^. The desirability of the response rises with *d* and its value varies between 0 and 1. However, studies using an integrated RSM and DF approach for parameters optimization for the heavy metals biosorption process are limited ^42^. Recently, Arumugam et al.^43^ demonstrated an integrated RSM and CSA-based approach to optimizing variables for the synthesis of a biolubricant. The parameters of water jet cutting process have been optimized using hybrid RSM–CSA ^44^. To the best of our knowledge, using RSM coupled with the CSA to optimize biosorption process parameters has not yet been explored in the literature. In this research, the hybrid RSM–DF method was utilized to optimize biosorption conditions to maximize Pb(II) elimination efficiency. Subsequently, the hybrid RSM–CSA platform was employed to achieve a globally optimal set. The anticipated operating conditions for the globally optimal solution were then confirmed through experimental results. In addition, kinetic data for Pb(II) biosorption were modeled using both pseudo-first order and pseudo-second order kinetic models. The validated platform was also examined for simultaneous biosorption processes from the mixture of heavy metals (i.e., Pb, Co, Cu, Cd, and Cr).

## Materials and methods

### Experiment

#### Microalgae species, growth conditions, and biomass

**Figure 1:**
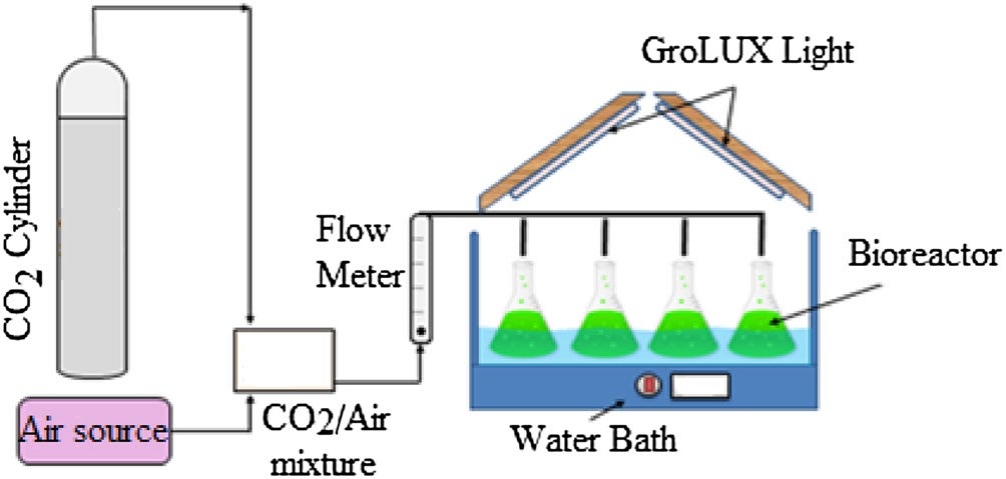
Experimental setup for cultivation of the *Chlorella kessleri.*

The microalgae (*C. kessleri* sp., UTEX-2229) sample was acquired from the University of Texas, USA. Bold’s basal medium was utilized for the microalgae growth culture. The microalgae were grown in bioreactors in Pyrex Erlenmeyer flasks at 30 °C, as shown in Fig. 1. The working volume was restricted to one litter with an initial algal concentration of 2.2 × 10^7^ cells mL^-1^. The bioreactors were placed on a bench with four Grolux fluorescents lightbulbs (average intensity: 65 µmol m^−2^ s^−1^) aligned in a wooden frame placed above the water bath. A combination of 4% CO_2_ and 96% air was delivered to the reactors. After nine days of cultivation, the microalgae biomass was collected and centrifuged, and subsequently washed with ddH_2_O. The biomass was then dried at 60 °C for 24 h in an oven. The dehydrated microalgae biomass was then ground and sieved to make particles around 1.0 mm in size, which were utilized for the heavy metals biosorption experiments.

#### Reagents and chemicals

All metal salts used in this study were analytical reagent grade and used without any further treatment. Pb(NO_3_)_2_ (purity ≥ 99%), Cd(NO_3_)_2_^.^ 4H_2_O (purity ≥ 98%), CuSO_4_^.^ 5H_2_O (purity ≥ 99%)_,_ Cr(NO_3_)_2_^.^ 9H_2_O (purity ~ 99%), and Co(NO_3_)_2_^.^ 6H_2_O (purity ≥ 98%) were purchased from Sigma-Aldrich. The reagents like hydrochloric acid (purity ~ 34–37%) and sodium hydroxide (extra pure) were purchased from Research Lab Fine Chemical Industries. 1 M NaOH or 1 M HCl was used to regulate the pH values of the initial solutions.

#### Preparation of solutions (Pb ion detection by AAS)

Stock solutions of 1,000 ppm for each heavy metal ion (i.e., Pb, Cd, Cu, Co, and Cr) were formulated separately by mixing the appropriate amount of individual metal salt in ddH_2_O and then diluting to 1 L in a volumetric flask with ddH_2_O. For instance, for Pb(NO_3_)_2_, 1.598 g was used to make 1,000 ppm stock solution. The detection of a given metal concentration in the experimental solution is based on its respective calibration curve. For the construction of Pb(II) calibration curve, six standard solutions of it were prepared by appropriate dilution of its stock solution. AAS equipment was then calibrated using aforementioned working standard solutions. The calibration curve for Pb(II) was prepared by plotting the absorbance as a function of Pb(II) ion concentration. Concentration of the Pb(II) ions present in the sample was then determined by reading its absorbance at a wavelength of 283.3 nm using AAS and comparing it on the standard calibration curve.

#### Characterization of biomass

The characterization of microalgae biomass (the adsorbent or biosorbent) was conducted using FTIR spectroscopy (Nicolet 6700 Thermo Fischer Scientific) and FESEM/EDX (Field Emission Scanning Electron microscope coupled with energy dispersive X-ray detector, Philips XL-30) analyses. In FTIR analysis, 2–3 mg of sample was evenly mixed with 0.4 g of KBr (potassium bromide). Afterwards, the spectra (infrared) of the samples were obtained in the range of 500–4,000 cm^-1^_._ For SEM/EDX analysis, the sample specimen was dispersed on a stub and then the data for SEM/EDX analyses were recorded.

#### Biosorption of heavy metals

The experiments on the biosorption of Pb(II) were conducted according to a batch scheme. In this regard, 100 mL glass flasks (acid washed) were filled with 50 mL of Pb(II) with adjusted concentrations (10 ppm) and pH values. An appropriate dose of microalgae biomass (the adsorbent) was then added and the mixture was shaken for 2 h at 130 rpm. The supernatants were then filtered and examined for Pb(II) concentration using an atomic absorption spectrophotometer (AA-6800, Shimadzu) at 283.3 nm.

For the simultaneous biosoprtion tests, all of the heavy metals (5 ppm each) were mixed into 50 mL DDH_2_O to obtain a single mixture. The optimal operating conditions for Pb(II) biosoprtion were considered for this study of the elimination of heavy metals ions. The supernatants were filtered and examined for ion concentrations using inductively coupled plasma emission spectroscopy (ICPE-9000, Shimadzu).

The percent levels of heavy metals removed by the microalgae biomass were determined by Eq. (2):

2 $$R = \frac{{C_{i} - C_{f} }}{{C_{i} }} \times 100$$

where R denotes the elimination efficiency, and C_i_ and C_f_ indicate the initial and final doses of heavy metals, respectively.

### Modelling and parameter optimization

#### Response surface methodology

The BBD, a response surface method, is a well-known statistical design of experiments (DoE) ^45–47^. It is a randomized design with a fewer number of experimental trials than some other methods. In this design, each factor has three levels (i.e., low, center, and high) that are symbolized with coded values (i.e., − 1, 0, and + 1, respectively). The expectation is that the optimum value is retained within the range.

The BBD is a potential design tool for fitting second-order polynomial equations. The general coded model equation is given in Eq. (3), as follows:

3 $${\text{y}} = \beta_{{0}} + \sum\limits_{i = 1}^{N} {\beta_{i} } x_{i} + \sum\limits_{i = 1}^{N} {\beta_{ii} } x_{i}^{2} + \sum\nolimits_{i < j}^{{}} {} \sum {\beta_{ij} } x_{i} x_{j} + \varepsilon$$

where *y* denote*s* the response, *x*_*i*_ denotes the coded parameters, *β*_*o*_ represents the intercept term, *β*_*i*_ denotes the linear effect, *β*_*ii*_ denotes the squared effect, *β*_*ij*_ denotes the interaction effect, and ɛ is the error term.

For statistical calculations, the relationship between coded and actual variables can be written as in Eq. (4).

4 $$coded\;value = \frac{{\left( {actual\;value - mean} \right)}}{half\;of\;range}$$

Table 1 shows the BBD matrix with a range and levels (both coded and actual) for three independent factors: pH, temperature, and biomass dose. Minitab (version 18) software was utilized for all statistical and graphyical analyses.

**Table 1:** Ranges and levels of experimental parameters. The coded values for each parameter are − 1, 0, or + 1 and the corresponding actual values are documented.

Parameter	Symbol	Range and level		
		− 1	0	+ 1
pH	A	4	6	8
Temperature (^o^C)	B	20	30	40
Biomass dose (g/L)	C	0.5	1.0	1.5

#### Parameter optimization with the hybrid RSM-DF

In metal removal efficiency, higher is better. Pursuing maximum removal, the DF (di, denoted as the *i*th targeted output) was integrated with RSM; this was obtained by creating a prediction profile plot using Minitab (version 18). The individual desirability (d) and composite desirability (D) are as follows:

5 $$d_{i} = \left\{ {\begin{array}{*{20}l} 0 \hfill & {y_{i} < L} \hfill \\ {\left( {\frac{{y_{i} - L}}{U - L}} \right)^{w} } \hfill & {L \le y_{i} \le U} \hfill \\ 1 \hfill & {y_{i} > U} \hfill \\ \end{array} } \right.$$

6 $$D = \left( {d_{1} \times d_{2} \times \ldots \times d_{n} } \right)^{\frac{1}{n}} = \left( {\mathop \prod \limits_{i = 1}^{n} d_{i} } \right)^{\frac{1}{n}}$$

where w denotes the weight, L and U are the lower and upper values, respectively, and y_i_ is the *i*th response. The response is treated unacceptable if d = 0 and ideal when d = 1 ^48^. The parameter of composite desirability (D) was used for multi-objective optimization. It is of note that for single-objective optimization both the desirability (d) and composite desirability values are the same.

#### Hybrid RSM–CSA

The regression model acquired from the RSM was adapted to assess the globally optimum combination of independent variables via the CSA. The computer code for the hybrid RSM–CSA was discussed in details elsewhere ^29,36,49–51^. Briefly, the steps can be written as follows:

1. Initialization of the parameters: The parameters involved flock size (N), flight length (fl), awareness probability (AP), and maximum iterations (${iter}_{max}$).
2. Initialization of position and memory: N crows take their positions in a random manner in the matrix of dimension (d) where d represents the decision variable. Each crow denotes an effective solution.
  7 $$Crows = \left[ {\begin{array}{*{20}c} {x_{1}^{1} x_{2}^{1} } & \cdots & {x_{d}^{1} } \\ \vdots & \ddots & \vdots \\ {x_{1}^{N} x_{2}^{N} } & \cdots & {x_{d}^{N} } \\ \end{array} } \right]$$
  8 $$Memory = \left[ {\begin{array}{*{20}c} {m_{1}^{1} m_{2}^{1} } & \cdots & {m_{d}^{1} } \\ \vdots & \ddots & \vdots \\ {m_{1}^{N} m_{2}^{N} } & \cdots & {m_{d}^{N} } \\ \end{array} } \right]$$
3. Assessing the fitness function: The initial position is investigated for each crow in the fitness function by d.
4. Creating a new position: The new position of Crow i is generated using Eq. (9) if Crow j notices that Crow i is observing her.
  9 $$x^{i, iter + 1} = \left\{ {\begin{array}{*{20}l} {x^{i, iter} + r_{i} \times fl^{i, iter} \times \left( {m^{j, iter} - x^{i, iter} } \right)\quad when\,\, r_{j} \ge AP^{j, iter} } \hfill \\ {{\text{a random position}}\quad otherwise} \hfill \\ \end{array} } \right.$$
5. Testing the effectiveness of the new position: The usefulness of the new position of each crow is tested.
6. Assessing the fitness function: The fitness function is assessed by the new position.
7. Revising the memory: The crow revises the memory by using Eq. (10):
  10 $$m^{i, iter + 1} = \left\{ {\begin{array}{*{20}l} {x^{i,iter + 1} {\text{if}} f\left( {x^{i, iter + 1} } \right) \;{\text{is}}\;{\text{better}}\;{\text{than}}\;f\left( {m^{i, iter} } \right)} \hfill \\ {m^{i,iter} \quad otherwise} \hfill \\ \end{array} } \right.$$

where $f(\bullet )$ denotes the objective function.

8. End benchmark: Steps (4) to (7) are repeated until ${iter}_{max}$ is achieved. The best positions of the memory provide the best objective function, as well as the solution to the factorial optimization problem.

### Investigation of adsorption kinetics

The surface adsorption processes control mechanism was investigated using adsorption kinetics. In this regard, the pseudo-first order and pseudo-second order models were expressed based on Eqs. (11) and (12), respectively.

11 $$log\left( {q_{e} - q_{t} } \right) = log\left( {q_{e} } \right) - \frac{{k_{1} }}{2.303}t$$

12 $$\frac{t}{{q_{t} }} = \frac{1}{{k_{2} q_{e}^{2} }} + \frac{1}{{q_{e} }}t$$

where k_1_ and k_2_ denote the first- and second-order rate constants, respectively, and ${q}_{t}$ denote the adsorption capacity at any time *t*, which can be expressed as:

13 $$q_{t} = \frac{{V\left( {C_{o} - C_{t} } \right)}}{m}$$

### Test of significance

Analysis of variance (ANOVA) was utilized to clarify the significance of linear, square and interaction terms based on probability values (*p* values). Generally, if *p* values < 0.05, the terms are statistically significant at 95% confidence level (α = 0.05) while *p* values < 0.1 indicating the terms are significant at 90% confidence level (α = 0.1). Contrarily, if *p* values > 0.1, the terms are insignificant. Minitab (version 18) statistical software was utilized for RSM model development and regression analysis, while MATLAB (2019a) was used for CSA-based analysis.

## Results and discussion

### Characterization of adsorbent

**Figure 2:**
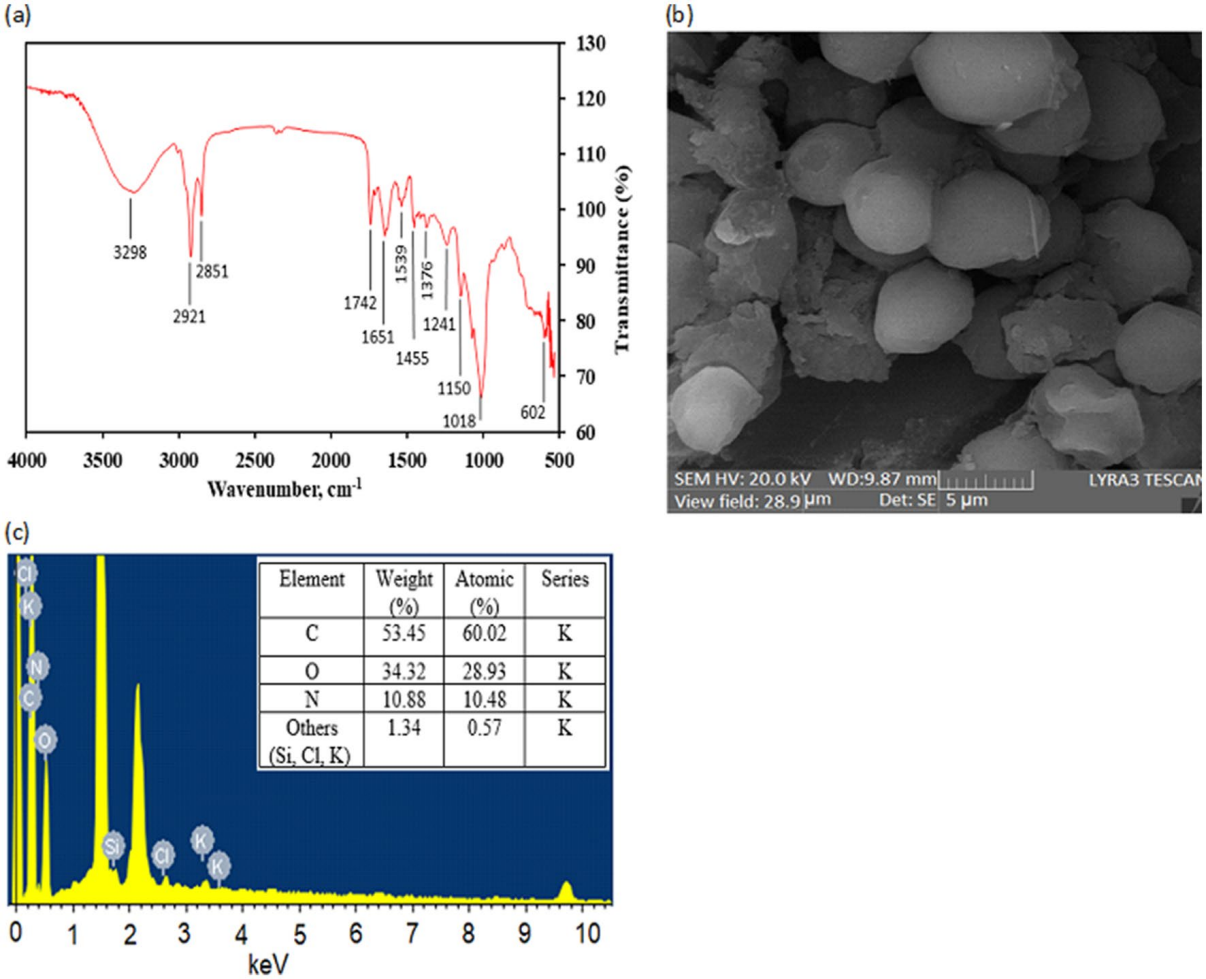
FTIR spectra (**a**), SEM micrograph (**b**), and EDX pattern (**c**) of microalgae adsorbent.

The microalgae biomass (the biosorbent or adsorbent) was characterized by using analytical techniques, FTIR and SEM/EDX. The FTIR spectrum of biomass was shown in Fig. 2a. It was evident that there were twelve different transmission bands over the wavenumber range of 4,000–500 cm^-1^. These bands were subjected to particular macromolecular groups such as lipids, proteins and carbohydrate. Each macromolecule contains specific functional group(s). Carbohydrates contain aldehyde and ketones groups while lipids contain mainly ester (both carboxylate and phosphate) and alcohol groups. Proteins encompass a wide range of functional groups which include thiols, alcohols, thioethers, carboxamides, carboxylic acids, and a range of basic groups.

The study band at 2,921 cm^-1^ and 2,851 cm^-1^ were due to the presence of “lipid band spectra” related to the symmetric C–H stretching of methylene. These bands associated with the presence of lipid were more intense than that of similar findings of other researcher^52^. The band at 3,298 cm^−1^ is owing to the O–H stretching. A strong peak at 1742 cm^-1^ as a result of C = O stretching of esters. In addition, another strong band at 1651 cm^-1^ is due to the C=O stretching of Amide I. The band at about 1539 cm^-1^ was Amide II band spectra mainly C–H and N–H stretching associated with proteins. The spectrum of 1,455 cm^-1^ associated with CH_2_ and CH_3_ bending of methyl and C–O stretching of carboxylic group. The protein band spectra were offered as medium pronounced band at about 1,241 cm^-1^ due to asymmetric stretching of phosphodiester P=O. The ‘carbohydrate band spectra’ was characterized by weak and medium features at about 1,150–839 cm^−1^ due to C–O and C–O–C stretching. The aforesaid bands spectra have indicated the existence of the key component in microalgae, namely lipid, protein, and carbohydrate. The results are in good agreement with several others^52–55^. The presence of these macromolecules expresses the existence of several functional groups (e.g., –OH, –COOH, –NH_2_, –CO), which are responsible for heavy metals ions biosorption.

The SEM micrograph for microalgae biomass is presented in Fig. 2b. The surface was very rough, cracked and damaged since the biomass was dried at 60 °C (24 h) and grounded. The EDX spectra of biomass sample is shown in Fig. 2c. It is noteworthy that EDX analysis enable to provide information about the composition of the adsorbent surface. The data (in terms of weight and atomic percentages) demonstrated the presence of C, O and N, which are the main components of cellular macromolecules.

### Statistical analysis of the Box–Behnken design

The experimental results (as shown in Table 2) were utilized to formulate the second-order polynomial model using multiple regression analysis and Minitab (version 18) software. The predictive regression model (see Eq. (14)) correlated the output response (Pb removal efficiency, Y) with three independent factors: initial solution pH (A), temperature (B), and microalgae biomass dose (C).

14 $$\begin{aligned} {\text{Y}} & = {94}.{65 } + {6}.{1}0{\text{1A }} - {2}.{18}0 {\text{B }} + { 7}.{238} {\text{C }} \\ & \quad - \, {11}.{24} {\text{A}}*{\text{A }} - {3}.{55} {\text{B}}*{\text{B }} - {2}.{84} {\text{C}}*{\text{C }} \\ & \quad - \, 0.{9}0{5} {\text{A}}*{\text{B }} - {2}.{4}0{1} {\text{A}}*{\text{C }} + 0.{713} {\text{B}}*{\text{C}} \\ \end{aligned}$$

**Table 2:** Experimental and predicted responses obtained from the BBD. The predicted outputs were calculated using model Eq. (9).

Run No	Coded value			Actual value			Response	
	A	B	C	A	B	C	Pb removal (%)	
							Y_exp_	Y_pre_
1	1	1	0	8	40	1	83.71	82.88
2	1	0	− 1	8	30	0.5	79.71	81.83
3	0	0	0	6	30	1	94.76	94.65
4	1	− 1	0	8	20	1	90.39	89.05
5	0	1	1	6	40	1.5	93.25	94.03
6	0	− 1	− 1	6	20	0.5	84.70	83.91
7	− 1	0	1	4	30	1.5	86.23	84.11
8	0	1	− 1	6	40	0.5	79.41	78.13
9	0	0	0	6	30	1	94.81	94.65
10	0	− 1	1	6	20	1.5	95.67	96.96
11	0	0	0	6	30	1	94.39	94.65
12	− 1	1	0	4	40	1	71.15	72.49
13	− 1	− 1	0	4	20	1	74.20	75.04
14	− 1	0	− 1	4	30	0.5	64.88	64.83
15	1	0	1	8	30	1.5	91.46	91.51

It should be noted that coded variables were utilized for all statistical analyses in order to retain the DoE as orthogonal. An orthogonal design offers choices for estimating model terms (each main effect and interaction) independently (i.e., bias-less) and thus creates the assessment straightforward. The predicted values for Pb removal efficiency obtained from Eq. (14) were also listed in Table 2.

**Figure 3:**
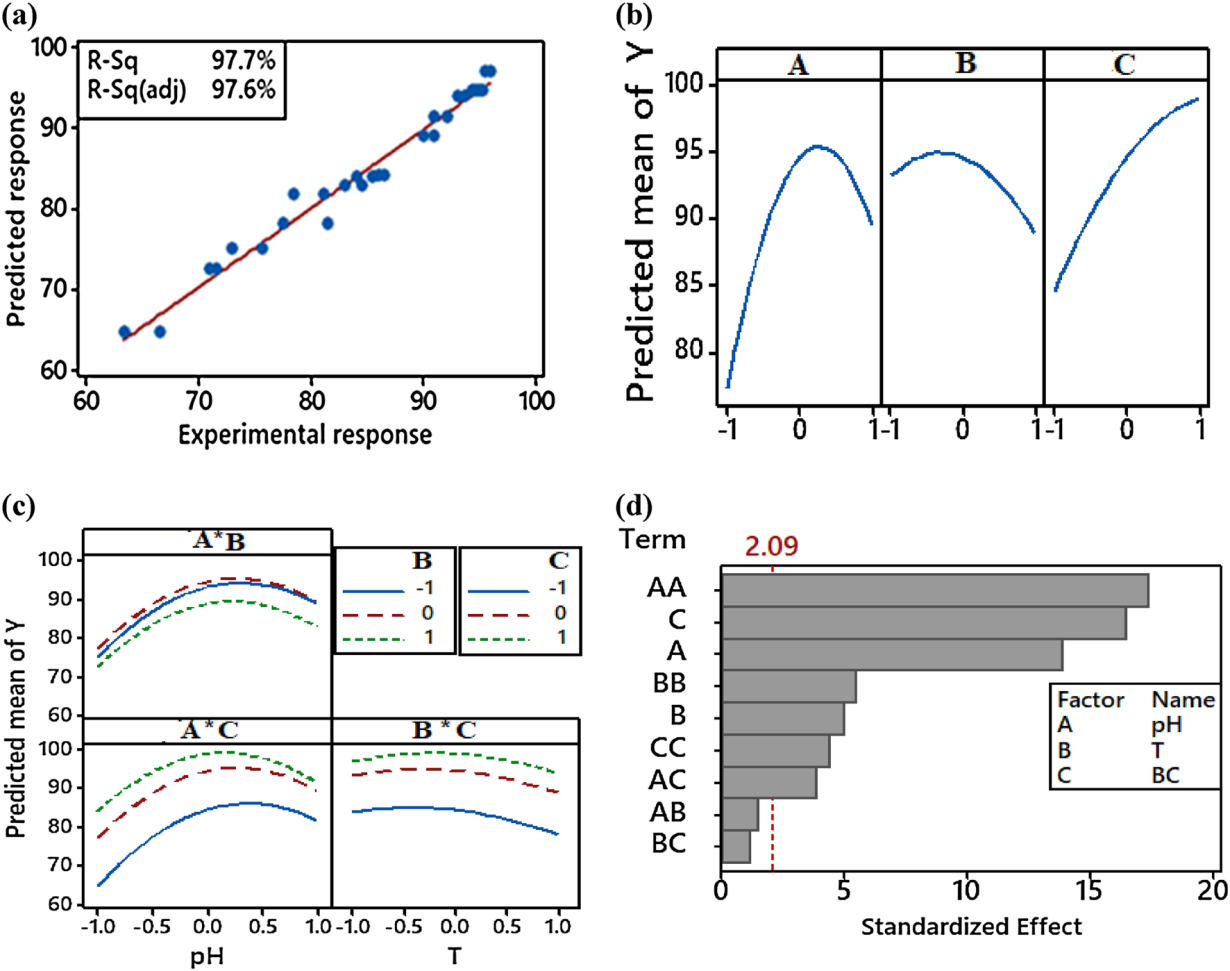
Fitted line (**a**), main effect (**b**), interaction (**c**), and Pareto (**d**) plots for the Pb(II) removal efficiency (Y). All independent parameters seemed important because various levels of each factor affected the output differently. Here, A, B and C represent pH, temperature and biomass dose, respectively.

In order to find the prediction capability of the model, a fitted line plot with coefficient of determination (R^2^) was generated which is shown in Fig. 3a. It expresses the relationship between the anticipated and experimental data for Pb(II) elimination efficiency. The high R^2^ (0.977) and adjusted R^2^ (0.976) values were very close, implying that the model did accurately forecast the response. To find the vital factor and interaction within the factors, main effect and interaction plots were generated (see Figs. 3b,c). Generally, the various levels of parameters differently impact the output. There is no main effect if the effect line is parallel to the horizontal axis. A sharply sloped line denotes greater importance in the main effect. Conversely, an interaction plot evaluates the interaction of independent factors; parallel lines suggest no interplay. The higher the value of the slope between the lines, the greater is the magnitude of interplay. The results in Fig. 3b show that all of the independent parameters (i.e., solution pH, temperature, and biomass dose) impacted the response (Y). The results also clearly demonstrated that all independent parameters were essential; however, the initial solution pH (A) was most important, then the biomass dosage (C), and after that temperature (B). Initial solution pH and temperature had both positive and negative effects, though biomass dose had only a positive effect on the output. Figure 3c shows the interaction of the parameters with one another. It is evident that there is no interaction or very low interaction between the parameters and response (Y).

To identify significant factors and their interactions on removal efficiency, a Pareto chart of the standardized effects is generated using Minitab software as shown in Fig. 3d. It is well known that if any bar of the linear, quadratic and interaction terms (A, B, C, A^2^, B^2^, C2, AB, AC, BC) passes the vertical red dotted line (average value of 2.09) as shown in the chart then it is significantly associated with the response. On the other hand, the bar that falls below the red dotted line is insignificant. It is clear from Fig. 3d that all independent factors (A = pH, B = T, C = biomass dose), square terms (A^2^ = pH^2^, B^2^ = T^2^, C^2^ = biomass dose^2^) and interaction term (AC = pH * biomass dose) are significantly associated with response except two interactions, AB (pH * T) and BC (T * biomass dose). The significant effects of all terms on Pb(II) removal efficiency decrease in the following order: A^2^ > C > A > B^2^ > B > C^2^. > AC.

To evaluate further, analysis of variance (ANOVA) table was generated which uses the F-statistic (or F-value) and the probability value (*p*). These values (F or *p* values) were calculated based on degree of freedom (DF), sum of square (SS) and mean square (MS). A high F-value generates a low *p* value when applying ANOVA analysis, and this p-value is used as the indicator to identify any term (model or parameter) as statistically either significant or not ^24,56^. Table 3 is the ANOVA table for Pb(II) removal efficiency, where the predictive model for Pb(II) removal efficiency was greatly significant since the model’s p-value was extremely low (*p* value = 0.000). The linear and quadratic model terms of A, B, C, A^2^, B^2^, and C^2^ were all significant at 95% confidence level (all *p* values < 0.050), while the interplay term AC was significant at 90% confidence level (*p* value < 0.1). The other interaction terms, AB and BC were found to be insignificant (all *p* values > 0.1). Overall, the ANOVA results were also in accordance with those presented in Pareto chart.

**Table 3:** ANOVA table for the regression model of Pb(II) biosoprtion.

Source	DF	Adj SS	Adj MS	F-Value	*p* value
Model	9	1,288.60	143.178	38.44	0.000
Linear	3	754.96	251.652	67.55	0.000
A	1	297.77	297.765	79.93	0.000
B	1	38.02	38.024	10.21	0.024
C	1	419.17	419.167	112.52	0.000
Square	3	505.27	168.423	45.21	0.000
A*A	1	466.51	466.508	125.23	0.000
B*B	1	46.59	46.595	12.51	0.017
C*C	1	29.87	29.867	8.02	0.037
2-Way Interaction	3	28.38	9.460	2.54	0.170
A*B	1	3.27	3.274	0.88	0.392
A*C	1	23.07	23.069	6.19	0.055
B*C	1	2.04	2.036	0.55	0.493
Error	5	18.63	3.725		
Lack-of-Fit	3	18.52	6.175	120.74	0.078
Pure Error	2	0.10	0.051		
Total	14	1,307.23			

DF, SS, MS, F-value, and *p* value denote the degrees of freedom, sum of square, mean of square, F-statistic, and probability value, respectively.

### Effects of input parameters on Pb(II) elimination efficiency

**Figure 4:**
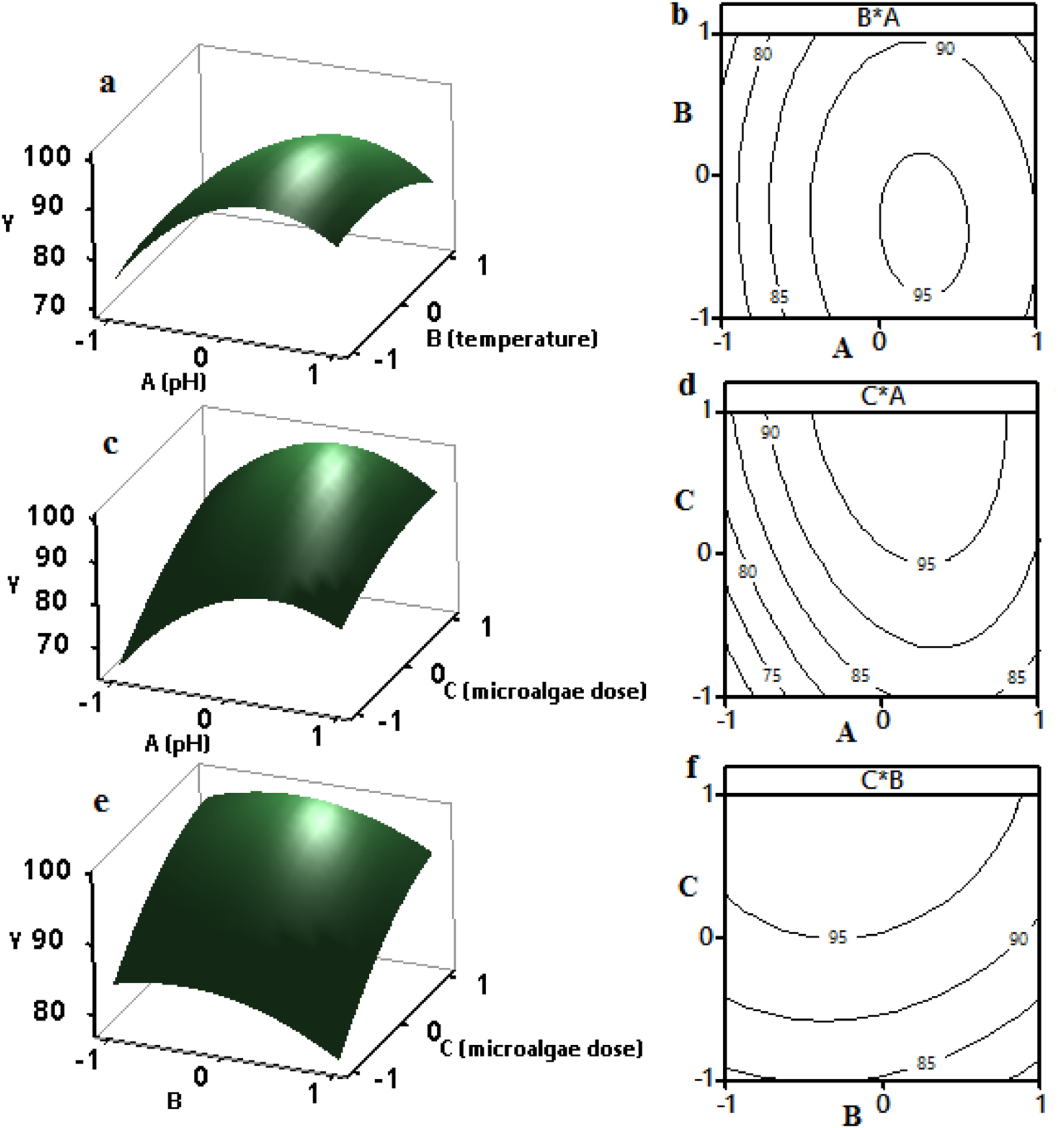
Response surface (**a**,**c**,**e**) and contour (**b**,**d**,**f**) plots indicating the effects of the parameters on Pb(II) removal efficiency (Y).

Figure 4 includes both 3D response surface (a, c, and e) and 2D contour (b, d, and f) figures to illustrate the impacts of several factors (i.e., pH, temperature, and microalgae biomass dose) on Pb(II) elimination efficiency (%). The figures were generated by utilizing two factors at the same time and holding the third factor fixed at the center point. Figure 4a,b show the Pb(II) removal efficiency (%) verses pH value and temperature with a constant biomass dose of 1 g L^−1^. The Pb(II) elimination efficiency was augmented by increasing the pH from 4 to 6 and temperature from 20 to 30 °C; then, it was decreased steadily. Both pH and temperature substantially affected Pb(II) removal efficiency. More than 95% efficiency was obtained with the approximate optimal set of a pH of 6 and temperature of 30 °C. At a very low pH, the cell surfaces were more positively charged due to a rise in H_3_O^+^, which may have deactivated the functional groups. Increasing the pH from a lower level to a below-neutral level enhanced the biosorption process, due to the rise in negative charge of the functional groups on the cell surfaces. At a pH value of more than 6, the precipitation of lead (II) occurred in the process, reducing the adsorption of the microalgae biomass. Usually, the adsorption process is exothermic, which favors a lower temperature. The biosorption efficiency began decreasing at a higher temperature, probably due to damage to the functional groups. In Fig. 4c,d, the Pb(II) elimination efficiency was augmented by increasing the pH from 4 to 6 and the microalgae biomass dose from 0.5 to 1.5 g L^−1^ while the temperature was kept constant at 30 °C. Similarly, in Fig. 4e,f, the Pb(II) elimination efficiency increased after elevating the temperature from 20 to 30 °C and microalgae dose from 0.5 to 1.5 g L^−1^, while the initial solution pH was kept constant at 6. From these figures, it is clear that the Pb(II) elimination efficiency increased after augmenting the microalgae biomass dose. The result is reasonable since the adsorption sites increased at higher algae biomass doses. These data support the conclusions of previous studies ^3,26,57,58^. The maximum Pb(II) removal efficiency of approximately 96% can be observed at the top of the surface plot and middle of the contour plot. However, it was difficult to obtain the exact optimum conditions using these plots. Thus, it was required to construct a response optimizer plot using a DF-based approach to finding the single optimal set.

### Parameter optimization using hybrid RSM-DF

**Figure 5:**
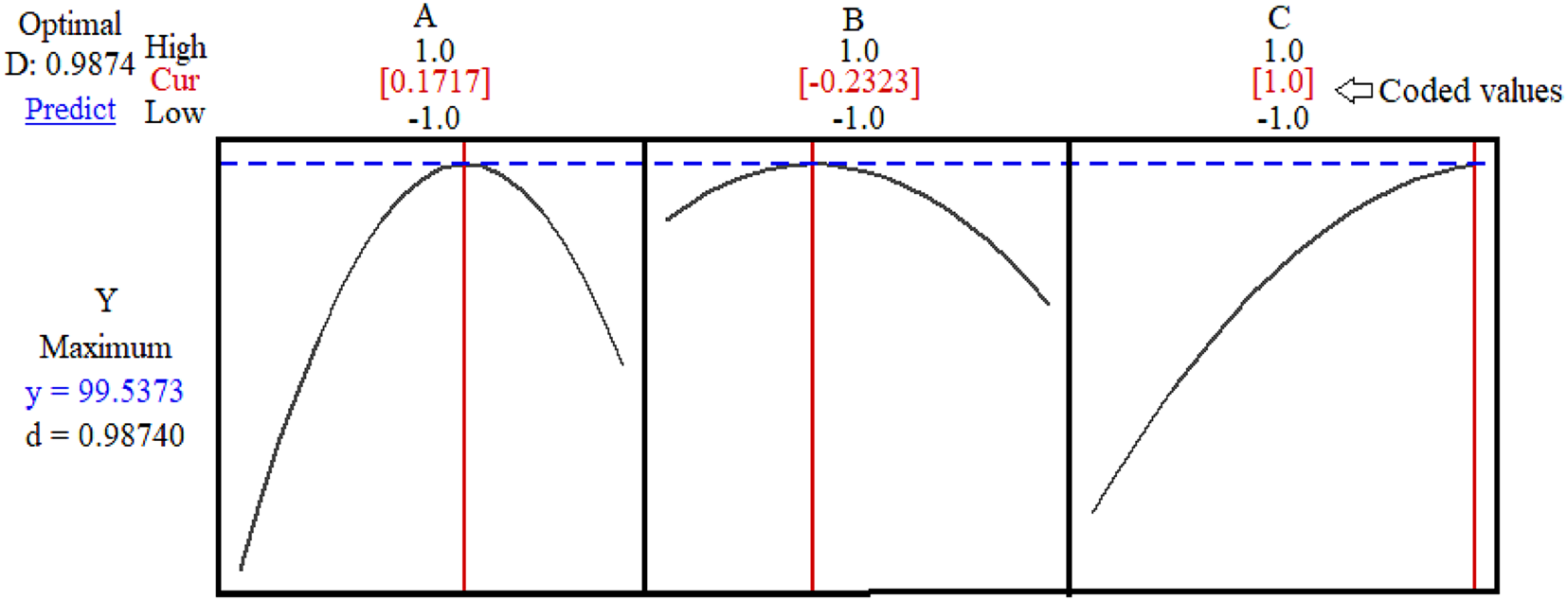
Prediction profile plot for Pb removal efficiency. The optimum coded values for A, B, and C were 0.1717, − 0.2323, and 1.0, respectively, and the removal efficiency was 99.5373%. The desirability (d) and composite desirability (D) values were the same: 0.9868.

An optimizer plot of the hybrid RSM–DF method was generated using Minitab (version 18), as depicted in Fig. 5. The optimum coded values for the initial solution pH, temperature, and microalgae biomass dose were 0.1717, − 0.2323, and + 1.0, respectively. These values are equivalent to a pH of 6.34, temperature of 27.67 °C, and microalgae biomass dose of 1.5 g L^−1^, respectively. The maximum Pb(II) elimination efficiency achieved under these optimum conditions was 99.537%. This set gave a desirability (d) or composite desirability (D) value of 0.9868, which is close to 1 and thus indicated that the optimal set was robust and reliable.

### Parameter optimization using the hybrid RSM–CSA platform

**Figure 6:**
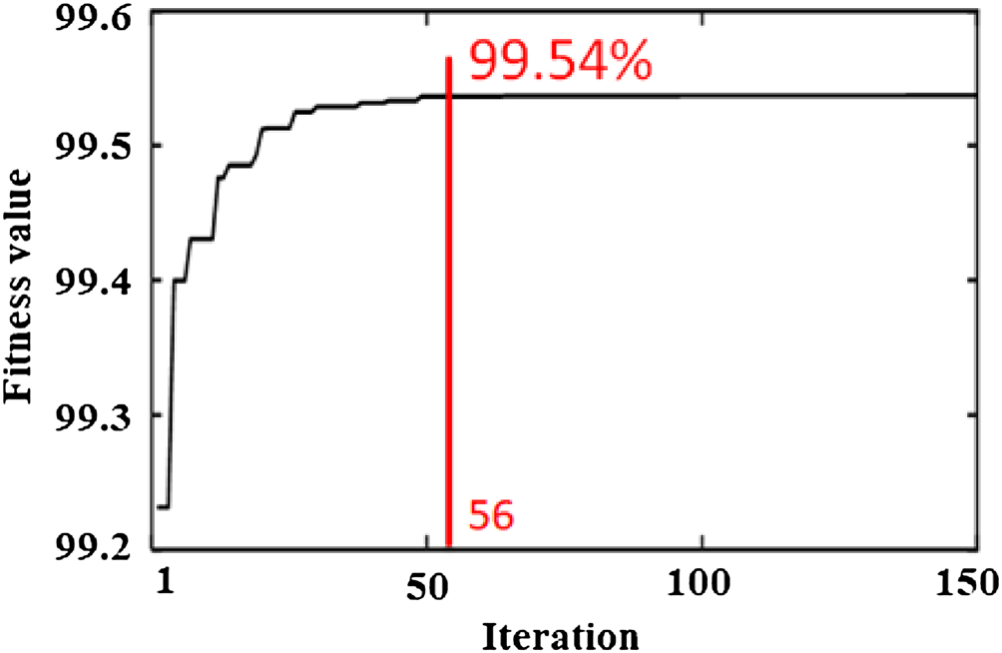
Convergence plot for Pb(II) removal efficiency along a number of iterations. The combined effects of all independent factors leaded to a stable output. After 56 iterations, a stable optimum point was achieved.

In this optimization technique, the fitted quadratic polynomial model acquired via the BBD (see Eq. 14) was treated as a fitness function and utilized to assess the global solution. The CSA code was generated using MATLAB 2019a software. A convergence plot of the fitness value versus the number of iteration for Pb(II) elimination efficiency is presented in Fig. 6*.* After 56 iterations, the Pb (II) elimination efficiency was almost consistent. The maximum Pb(II) elimination efficiency of 99.54% was achieved with coded values for initial solution pH, temperature, and microalgae dose of 0.1739, − 0.2288, and 0.9999, respectively, which are equivalent to 6.34, 27.7 °C and 1.49 g L^−1^, respectively.

The initial solution pH, temperature, and microalgae dose obtained using the RSM–CSA approach gave the same values as did the RSM–DF technique, indicating that the RSM–DF optimization was global in this optimization process. Since the CSA depends on random formation, a little fluctuation in the response (i.e., removal efficiency) was seen for each run of the optimal set ^59^. Hence, 50 experiments were operated separately, and the mean and standard deviation of the data were obtained, as given in Table 4. The very low standard deviation of 1.4 × 10^–5^ demonstrates that the optimal point achieved by the CSA was powerful and reliable. For verification, duplicate experiments were conducted with this optimum set. The experimental results show that 97.37% of the Pb(II) was discarded by the microalgae at these conditions. The experimental removal efficiency was very close to the model’s prediction (with an error rate of less than 5%), indicating the accuracy of the model. Therefore, the optimized conditions achieved by both the local (RSM–DF) and global (RSM–CSA) hybrid methods for Pb(II) removal were verified.

**Table 4:** Statistical results obtained by CSA using 150 iterations. In these iterations, the best, worst, mean, and standard deviation of the Pb removal efficiency are presented.

Best	Worst	Mean	Std
99.537002	99.536932	99.536982	1.4 × 10^–5^

### Biosoprtion kinetics

**Figure 7:**
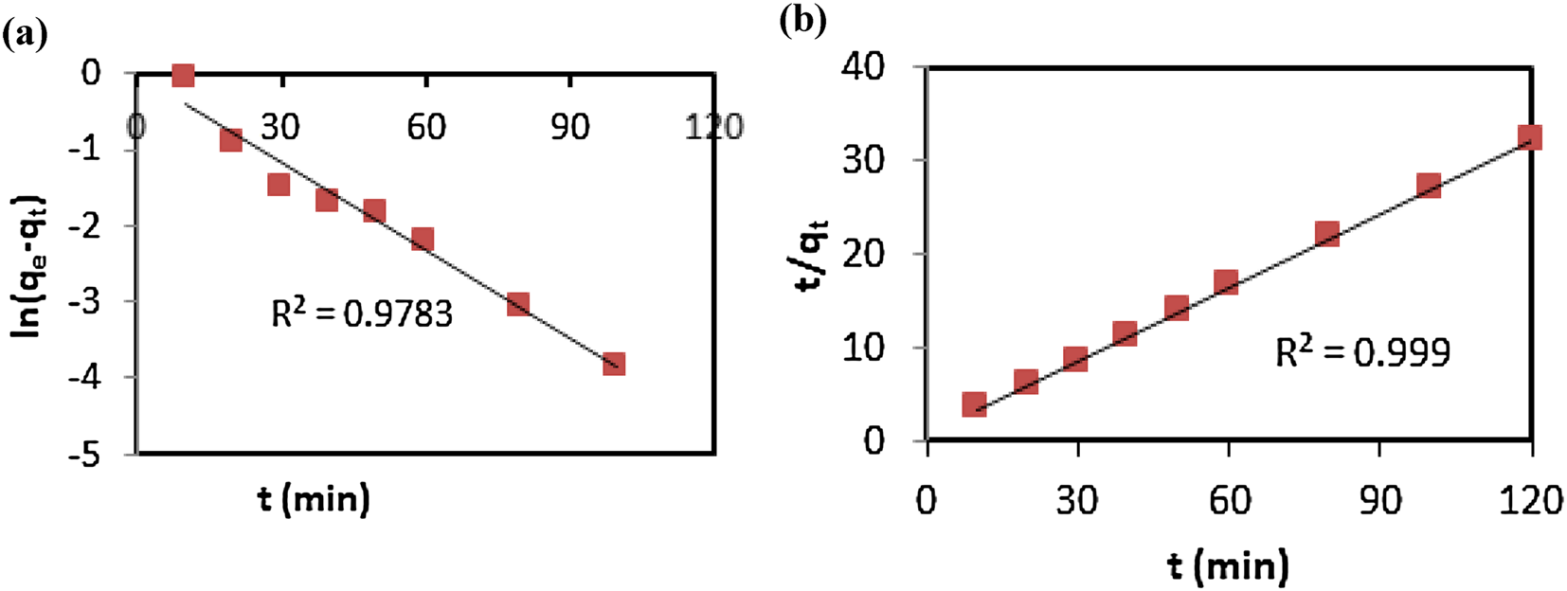
Different kinetic model plots using experimental data: pseudo-first order model (**a**) and pseudo-second order model (**b**).

Two kinetic models (e.g., pseudo-first, pseudo-second order) were utilized to investigate Pb(II) biosorption kinetics, which is shown in supplementary Figure S1. From this figure, it is evident that the biosoprtion capacity increased rapidly within the first 20 min; almost all of the biosoprtion occurred in that period. After 20 min, the biosoprtion capacity increased slowly and plateaued at around 100 min. The maximum biosoprtion capacity (q_e,exp_) was found to be 3.727 mg g^−1^. Figure 7 shows the experimental data fitted with the pseudo-first order (Fig. 7a) and pseudo-second order (Fig. 7b) kinetics models. The R^2^ value for the pseudo-second order kinetic model was bigger (R^2^ = 0.999) in comparison to the pseudo-first order model (R^2^ = 0.9783). The parameters of the two models are documented in Table 5. The value of q_e,calc_ calculated from the pseudo-second order kinetic model was very close to the experimental value (q_e,exp_). The overall results indicated that the superior performance was observed in pseudo-second order kinetic model in interpreting the biosorption kinetics of Pb(II) in response to green microalgae biomass. These results support with those of previous studies ^13,60–62^.

**Table 5:** Kinetic parameters for biosoprtion of Pb(II) by the microalgae biomass.

Kinetic model	q_e.exp_ (mg/g)	q_e_._calc_ (mg/g)	Rate constant
Pseudo first order	3.727	0.966	0.0879 (min^−1^)
Pseudo second order	3.727	3.782	0.1148 (g mg^−1^ min^−1^)

Conditions: pH of 6.3, temperature of 27.7 °C, and biomass dosage of 1.5 g/L.

### Comparison with other biosorbents (green microalgae biomass)

A comparison between the removal efficiency of Pb(II) in this work and others found in the literature is presented in Table 6. The percentage removal efficiency of Pb(II) found in this work is significantly higher than reported for other green microalgae biomass (biosorbents) except that of *Chlorella vulgaris*. Thus, the comparison of removal efficiency shows that the microalgae *C. kessleri* is an effective biosorbent to eliminate the lead metal ion from wastewater.

**Table 6:** Removal efficiency of Pb(II) by various biosorbents (green microalgae biomass).

Biosrbent (green microalgae biomass)	Removal efficiency (%) of Pb(II)	Literature
*Spirogyra* sp.	80	^63^
*Oedogonium* sp.	70	^64^
*Nostoc* sp.	78	^64^
*Spirulina platensis*	84	^65^
*Spirulina* sp.	91	^66^
*Spirulina Maxima*	83	^67^
*Spirulina platensis*	90	^68^
*Chlorella vulgaris*	99.4	^69^
*Chlorella sorokiniana*	91	^70^
*Chlorella kessleri*	97.1	^This study^

### Simultaneous biosorption from a mixture of heavy metals

**Figure 8:**
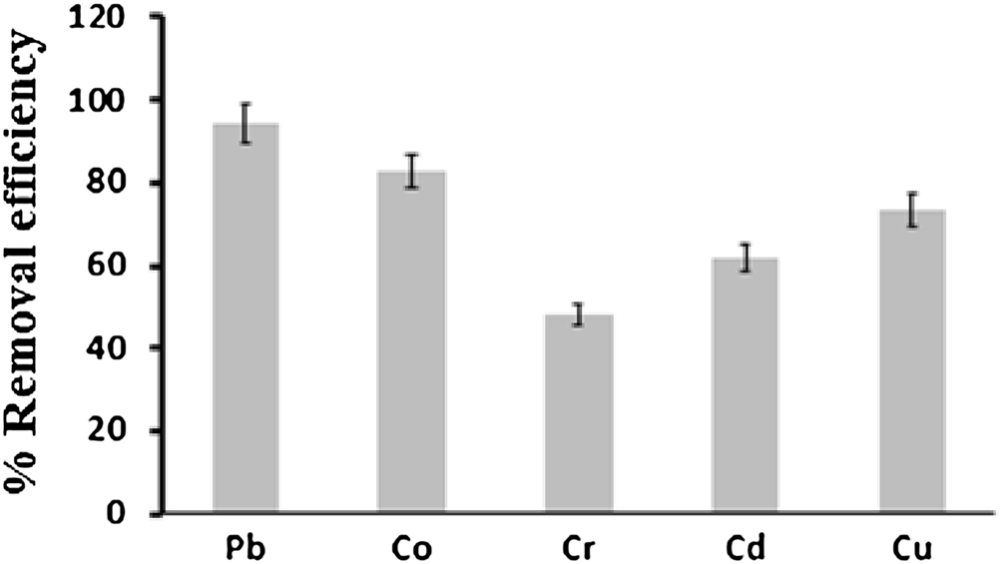
Efficiency of removing a mixture of heavy metals using microalgae for the biosorption process. Conditions were: pH of 6.3, temperature of 27.7 °C, and biomass dosage of 1.5 g/L.

The simultaneous biosorption process for a mixture of heavy metals was examined in this study to test the utility of the biosorption process of microalgae for industrial cases, since most industrial wastewater contains multiple heavy metals ions. Figure 8 shows the ion removal efficiency for a mixture of heavy metals. The data demonstrated that the elimination efficiency for Pb was high (˃ 90%), while the removal efficiencies for Cr and Cd were low (around 50–60%). This discrepancy could be due to each metal’s electronegativity or availability near the active sites of the biosorbent. The electronegativities of Pb, Cu, Co, Cd, and Cr were 2.33, 1.9, 1.88, 1.69, and 1.66, respectively. Therefore, the maximum removal efficiency was found for the Pb ions, while the minimum removal efficiency was observed for the Cr ions. The electronegativities for Cu and Co were very close and the removal efficiencies were comparable. The removal efficiencies of the metal ions increased in the following order: Cr < Cd < Cu < Co < Pb. This study proves that the adsorption of heavy metals by active microalgae sites varied due to changes in the electronegativity of the heavy metals ions. However, further investigation is necessary to optimize the simultaneous biosoprtion process.

## Conclusion

This research examined the performance of a microalgae biomass for efficiently eliminating heavy metals from an aqueous solution. Initially, the adsorbent (microalgae biomass) was characterized using FTIR, and SEM/XRD analyses. To maximize Pb(II) removal efficiency, an RSM-based DF was utilized to optimize several Pb(II) removal process factors, including the initial solution pH, operating temperature, and microalgae biomass dose. The second-order model developed was interpreted with ANOVA in terms of significant factors and their interactions. The optimal set was an initial solution pH of 6.34, temperature of 27.67 °C, and biomass dose of 1.5 g L^−1^; this set provided a maximum Pb(II) removal efficiency of 99.537%. Next, the performance of hybrid RSM-CSA optimization was studied, and the data confirmed the RSM-DF results. The data predicted were verified with experimental values, with an error rate of < 5%. Thus, the optimal operating set based on a nature-motivated metaheuristic algorithm was both powerful and reliable. In addition, the results demonstrated that the pseudo-second order model was the best for Pb(II) biosoprtion on the surface of the microalgae. Finally, the bioremediation of ions from a mixture of metals was also successfully accomplished. The elimination efficiency of each heavy metal varied due to differences in the electronegative interactions. Overall, this successful application of the hybrid RSM–CSA approach was the first to accomplish microalgae-based elimination of heavy metals from a synthetic wastewater solution and is applicable for industrial wastewater treatment. Further, this hybrid platform could be utilized as a pivotal tool for exploring the impacts of parameters on using microalgae biomass for the removal of multiple heavy metals from actual samples.

## Supplementary information

Supplementary Figure S1.

## Abbreviations

RSM: Response surface methodology
BBD: Box–Behnken design
A: Initial solution pH
B: Temperature (^o^C)
C: Biomass dose
ANOVA: Analysis of variance
SS: Sum of square
MS: Mean of square
DF: Degree of freedom
Y: Pb(II) removal efficiency
d: Desirability
D: Composite desirability
CSA: Crow search algorithm
AP: Awareness probability
fl: Flight length
N: Flock size
M: Molar concentration (mol l^-1^)
