# Supplementary material for: Experimental study and parameters optimization of microalgae based heavy metals removal process using a hybrid response surface methodology-crow search algorithm
Source: Sci Rep. 2020 Sep 15;10:15068. doi: 10.1038/s41598-020-72236-8 (PMC7493913; doi:10.1038/s41598-020-72236-8)
Supplement: Supplementary file 1 — Supplementary Figure S1. [file 41598_2020_72236_MOESM1_ESM.docx]

**Experimental Study and Parameters Optimization of Microalgae based Heavy Metals Removal Process using a Hybrid Response Surface Methodology-Crow Search Algorithm**

N. Sultana^1^, S. M. Zakir Hossain^2*^, M. Ezzuding Mohammed^2^, M. F. Irfan^2^, B. Haq^3^, M. O. Faruque^4^, S. A. Razzak^4^, M. M. Hossain^4^

^1^Department of Computer Science, College of Computer Science and Information Technology, Imam Abdulrahman Bin Faisal University, Saudi Arabia

^2^Department of Chemical Engineering, College of Engineering, University of Bahrain, Kingdom of Bahrain

**^3^**Department of Petroleum Engineering, King Fahd University of Petroleum & Minerals, Saudi Arabia

**^4^**Department of Chemical Engineering, King Fahd University of Petroleum & Minerals, Saudi Arabia

*Corresponding author detail:

Tel: (973) 1787-6374

Fax: (973) 1768-0935

E-mail: [zhossain@uob.edu.bh](mailto:zhossain@uob.edu.bh)

**
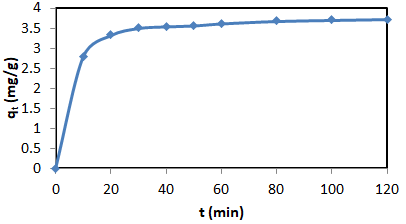
**

Supplementary **Figure S1.**
